# Reduction of Space Groups to Subgroups by Homogeneous Strain

**DOI:** 10.6028/jres.067A.042

**Published:** 1963-10-01

**Authors:** H. S. Peiser, J. B. Wachtman, R. W. Dickson

## Abstract

It is assumed that the symmetry elements possessed by a strained crystal will be those common to the unstrained crystal and to the macroscopic state of strain. This principle has been applied to show all of the possible subgroups to which a given space group can be lowered by homogeneous strain for all of the 230 crystallographic space groups.

## 1. Introduction

The symmetry of strained crystals is important in the following considerations:

1. The presence or absence of internal friction resulting from the motion of point defects in a crystal can depend upon whether or not the initially equivalent sites accessible to the defect are in equivalent in the strained crystal [*1, 2, 3, 4*].^1^ An isolated point defect, such as a vacancy, will occupy any one of a set of atomic sites extending throughout the crystal with equal probability in the absence of strain; if the set splits into inequivalent subsets under strain, internal friction will occur when the frequency of an alternating strain is approximately equal to the jump frequency for the point defect.
2. Any tensor property of a crystal, such as piezoelectricity, depends primarily upon the symmetry of the unstrained crystal, but additional tensor components may be introduced by straining the crystal in such a way as to change its symmetry.
3. A classification of the types of polymorphism of crystals has been proposed by M. J. Buerger [*5*]; in some categories no bonds are broken but only a symmetry change takes place. Some structural changes can be induced by homogeneous strain.
4. Introduction into crystals of impurity atoms is accompanied by strain which may lower the symmetry. Such symmetry inversions are well known and properly regarded as phase transformations. At the same time it is tempting to broaden the use of the term solid solution to include inversions in which some crystallographic parameter measuring the departure from higher symmetry is a continuous and increasing function of impurity concentration (in a range including zero concentration). If this function is linear to first approximation the behavior would be a simple extension of Vegard’s Law. In any event this phenomenon would be limited to symmetry inversions in conformity with strict postulates applying to continuous transformations by strain, that is second-order transformations in the strict sense used by Landau and Lifshitz as discussed by Dimmock [*6*].
5. Large strain fields exist near dislocations and accompanying symmetry changes may be associated with large local variations in physical properties such as enhanced diffusion near a dislocation.
6. Strain-induced alteration of symmetry may cause change in electron-spin resonance [*7*] or infrared absorption [*8*]; measurement of these changes may give information on the type of site occupied by a given point defect.
7. The lowering of the symmetry of a crystal of doubtful point group may make a more definitive test available for determining the class of the unstrained crystal.

There are probably other effects associated with strain-induced lowering of symmetry, but the aim of the present paper is confined to the solution of the formal problem of the possible lowering of space-group symmetry by homogeneous strain.

The usual concept of homogeneous strain can be extended downward in scale to describe accurately the change of shape of the unit cell, but it will not in general describe the atomic movements within the cell. However, the present considerations involve only the symmetry of the crystal structure. The atoms within the unit cell need not move as if they were suspended in a continuous medium undergoing homogeneous strain. It is only required that their movements be consistent with the symmetry of the macroscopic strain.

## 2. Scope of Present Paper

In general, a crystal may be subjected to a stress that causes a strain which need not be homogeneous and which may have a special amplitude. This paper is, however, restricted to the consideration of homogeneous strain of arbitrary amplitude.

It may seem more natural to consider an applied stress as an imposed condition rather than a state of strain. It makes no difference to the present argument which is taken as the imposed condition because only the symmetry elements are significant. We defer discussion of this point until Curie’s principle is taken up in the next section.

Strain gradients may be important in some processes, such as Nabarro-Herring creep [*9, 10*] but the effect of a strain gradient must be superimposed on the effect of the average value of the strain in the region in which the physical process under consideration takes place. Creep involves transport of matter over macroscopic distances and may be associated with a strain gradient even though the average strain is zero. The properties listed in the introduction are, however, more likely to depend on average strain over an appropriate volume than on strain gradient because the smallest volume of crystal which can be used for discussion of these properties is comparable to the unit cell of the crystal. Accordingly, attention is restricted to homogeneous strain in this paper although it is recognized that symmetry changes caused by strain gradients may be significant for some physical properties.

One may specialize a strain with respect to orientation or with respect to magnitude, but the former is of more general interest. Thus one might apply a tensile stress to a tetragonal crystal in such a way as to lower it to orthorhombic symmetry and then look for effects on physical properties. Such an experiment would require only a knowledge of crystal orientation. Alternatively, one might apply a tensile stress along the unique axis of a tetragonal crystal and choose its amplitude such that this axis is made equal to the other two thus imposing a pseudocubic character on the crystal. Such an experiment requires a knowledge not only of crystal orientation but also of lattice parameters and elastic constants. This second type of experiment seems of limited interest and we restrict consideration in this paper to strain which may be specialized with respect to orientation, but not with respect to magnitude.

A strained crystal may undergo a phase change and the space group of the new polymorph need not necessarily be symmetry related to the starting crystal. We therefore specifically exempt phase changes from these considerations except those introduced by a continuous process such as those noted in the introduction.

## 3. Working Principle and Uniqueness of Symmetry Reduction

The components of homogeneous macroscopic strain form a tensor of second rank conveniently represented by a triaxial ellipsoid of symmetry point group mmm. When any two of its major axes are equal the ellipsoid acquires rotational symmetry about the third major axis. When all three major ellipsoid axes are equal it becomes a sphere. In our study of symmetry of strained crystals it is necessary to consider all kinds of possible orientations of the strain ellipsoid relative to the crystal symmetry elements.

We assume that homogeneously strained crystals will have all the symmetry elements common to the unstrained crystal and to the macroscopic strain, but will possess no other symmetry elements. It is an extension of Curie’s principle [*11*] to include space-group as well as point-group operations. Curie’s principle has been discussed by Shubnikov [*12*] and Koptsik [*13*].

Homogeneous strain possesses all possible translational symmetry elements and therefore preserves all lattice translations, all glide planes parallel to mirror planes of the strain, and screw axes parallel to rotation axes of equal or higher order.

In applying this principle to specific groups it is convenient to characterize strain by its point group. A situation sometimes arises in which the strain has a mirror plane parallel to a glide plane in the space group of the unstrained crystal. We assume the glide plane remains in the strained crystal. The same assumption is made regarding the retention of an *n*-fold screw axis in the crystal when it is parallel to an *n*-fold rotation axis in the strain. The process of taking symmetry operations common to the macroscopic strain and to the unstrained crystal must be understood to have this meaning. This situation is a consequence of the well known fact that a crystal may have glide planes and screw axes corresponding to the mirror planes and rotation axes of its point group.

The process of finding the space group of a homogeneously strained crystal can then be carried out in either of two ways. First, the elements strictly common to the point group of the unstrained crystal and to the point group of the strain can be found to give the point group of the strained crystal. There will in general be several space groups corresponding to this final point group. The correct space group will be the one which not only belongs to the final point group but which is also a subgroup of the initial space group. The subgroups of the space groups are listed in the Internationale Tabellen zur Bestimmung von Kristallstrukturen [*14*], Second, one can bypass consideration of the point group of the crystal and work directly with its space group, taking its elements in common with the point group of the strain in the sense explained in the last paragraph. The writers have used both methods as a check and a few misprints in the Internationale Tabellen were found.

One can now see that it makes no difference to the present work whether stress or strain is used as the imposed condition because in either case only the point-group symmetry is involved.

The reduction of the symmetry of a given crystal by a given strain with specified orientation is unique. The second process, described above, for finding the final space group from the initial space group and the strain is clearly unique. A given symmetry operation in the point group of the strain either does or does not have a corresponding operation in the space group of the unstrained crystal; the number of operations in this point group is finite and small so that every one can be examined to give a definite, unique answer for the final space group. The association of crystal with space group might be questioned but this is also unique. In particular, it will be possible to find a general position (characterized by point symmetry 1 and by a specific arrangement of the atoms around the position) which is acted on by every element of the space group so that in every primitive cell there are a number *N*, equal to the order of the point group, of identical, distinct positions. Application of the strain cannot raise the point symmetry, but can only remove some of the symmetry operations originally relating the *N* positions. Thus, the original set of *N* positions splits into an integral number, *N/n*, of subsets each containing *n* equivalent general positions in the strained crystal where *n* is the order of the point group of a strained crystal. A set of general positions defines a space group so the association of the strained crystal with a space group is unique.

An alternate approach to the lowering of crystal symmetry using matrix representation of the symmetry operations has been discussed by Ordway [*15*].

## 4. Results

**Figure 1:**
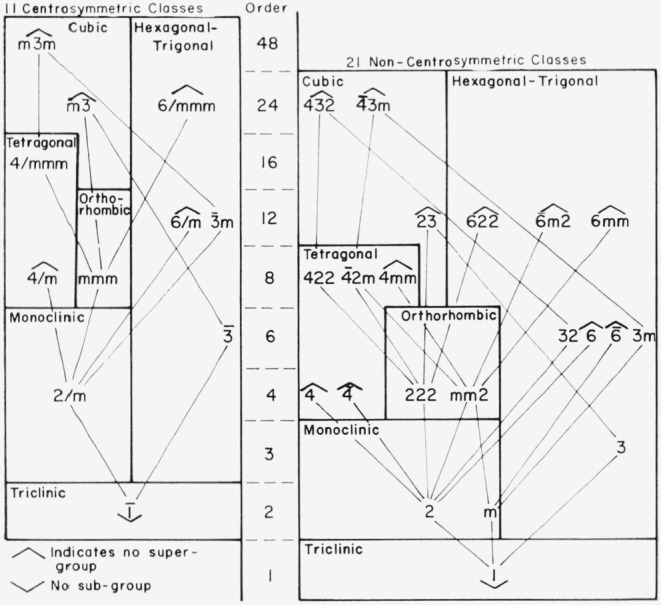
Reduction of point groups to subgroups by homogeneous strain. It is a necessary condition that a subgroup belong to a different crystal system than the corresponding supergroup. The point group designations are those of the International Tables [*16*].

The reduction scheme for crystallographic point groups is shown in figure 1. This result has been given before and the rules for its construction have been discussed [*1*, *3*]. It is shown here for completeness and to illustrate the important fact, not previously discussed, that some of the point-group reductions can be made in two or more crystallographically equivalent ways. Thus, the reduction from 4/mmm can be made either by retaining the (100), (010), and (001) mirror planes or by retaining the (110), $\left(1\bar{1}0\right)$, and (001) mirror planes depending on the orientation of the strain. Thus, two or more space-group reductions, corresponding to two or more strain orientations, can be associated with a given point-group reduction.

A given strain orientation (relative to the crystal) that is not represented by a single tieline can be considered as an equivalent sum of strains successively lowering the symmetry along two or more tielines.

**Figure 2:**
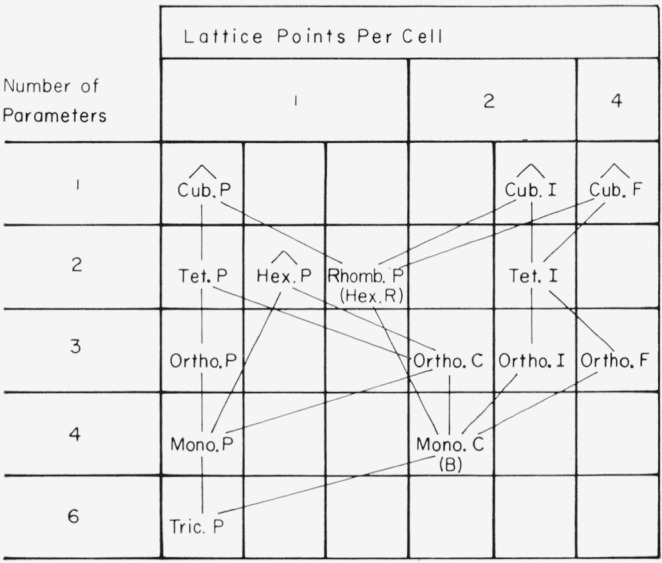
Reduction of Bravais-lattice symmetry by homogeneous strain. It is a necessary condition that a lattice go to one with a larger number of parameters. P means primitive, R stands for the compound hexagonal lattice derivable from the primitive thombohedral, C means centered on the C face, I means body centered, and F means centered on all faces.

The Bravais-lattice reduction scheme for homogeneous strain is shown in figure 2. Here a given starting lattice must go to a lattice with a larger number of parameters so that only transitions downward along the tielines shown are possible under homogeneous strain.

**Figure 3:**
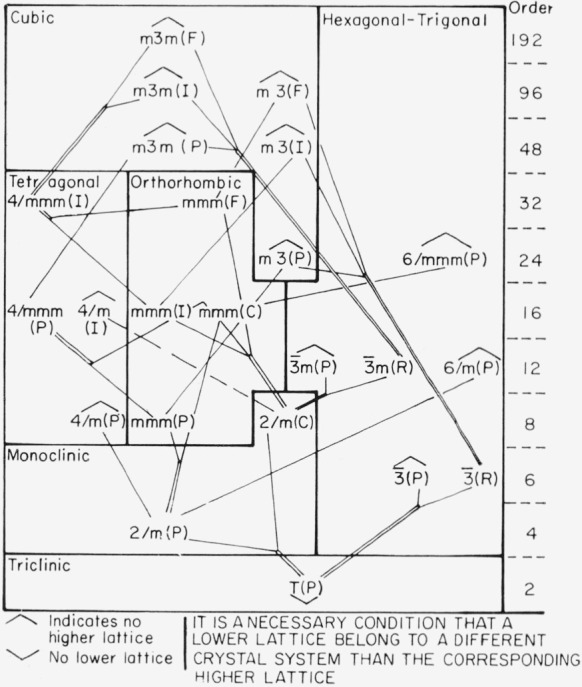
Reduction of point-group—Bravais-lattice combination by homogeneous strain for centrosymmetric point groups.

The results in figure 2 can be combined with the results for centrosymmetric point groups in figure 1 to give the reduction scheme for the combined centrosymmetric point groups—Bravais lattices shown in figure 3. This chart shows that certain possibilities are ruled out by the Bravais-lattice reduction scheme. Thus, a space group associated with point group m3m and with a face-centered lattice can go to a space group associated with point group 4/mmm but only to one with a body-centered lattice, not to one with a primitive lattice. These restrictions are automatically obeyed when either of the two processes described earlier is used to obtain a reduced space group. The relations in figure 3 do provide a useful partial check on such results. The writers have constructed a chart similar to figure 3 for the noncentrosymmetric point groups combined with the Bravais lattices. The construction is easy, but slightly tedious, and the results do not warrant publication as a step in the process of checking the final product, the space-group charts.

**Figure 4:**
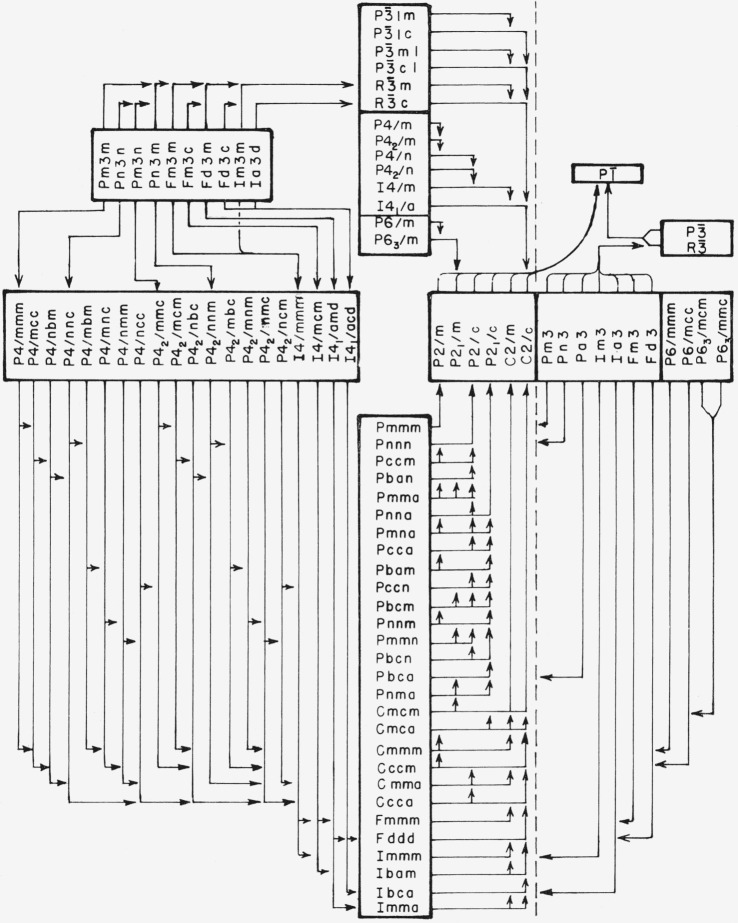
Reduction of centrosymmetric space groups by homogeneous strain. The notation of the International Tables [*16*] is used. Space groups associated with a single point group are enclosed in boxes. Arrows are drawn from starting space group to subgroup reached by homogeneous strain. A space group having no entering arrow cannot be obtained by homogeneous strain from a higher symmetry space group.

**Figure 5:**
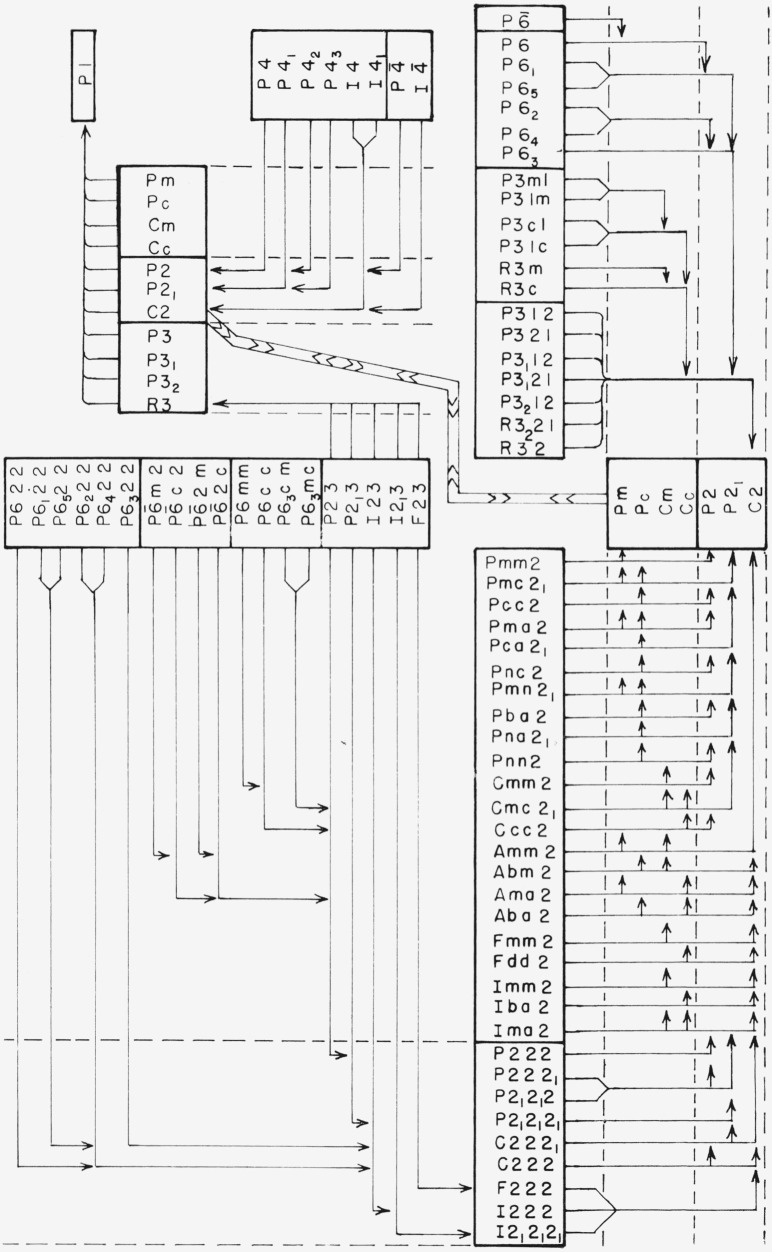

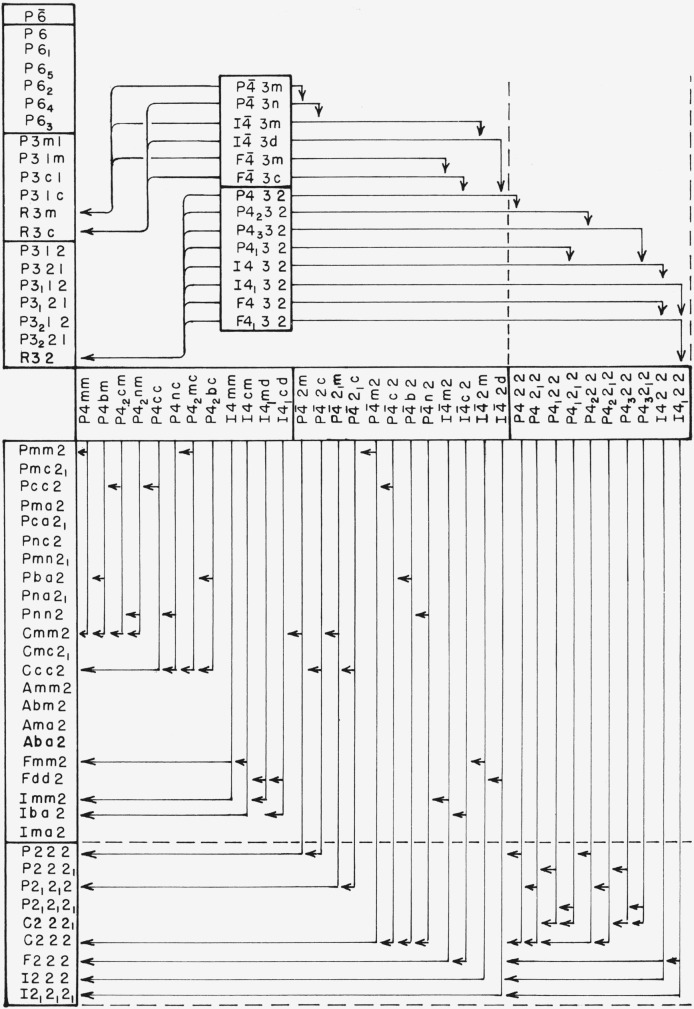
Reduction of noncentrosymmetric space groups by homogeneous strain. The vertical column, consisting of space groups associated with point groups 6, $\bar{6}$, 3m, 32 mm2, and 222, has been repeated on page 400 to provide sufficient space for entry and exit of arrows. The space groups associated with point groups m and 2 have been shown twice on the left hand page for the same reason. The text gives an example of the use of this chart.

The final results are shown in figure 4, for centrosymmetric space groups, and figure 5 for noncentrosymmetric space groups. This division into two charts is possible because homogeneous strain is centrosymmetric and cannot change the space-group property of being centrosymmetric or noncentrosymmetric.

As in the scheme shown in figure 1 a plane of symmetry on strain transformation is preserved only if it is perpendicular to one of the principal axes of the strain ellipsoid for any permissible choice of principal axes. A symmetry axis can be retained only if it is parallel to one of these principal axes of the ellipsoid. An axis of order higher than two can be retained only if it is perpendicular to the circular section of an ellipsoid of revolution. A plane of symmetry parallel or a two-fold axis perpendicular to an axis of order higher than two cannot be removed without simultaneous loss of the higher-order axis.

To illustrate the use of these charts, we discuss the reduction of group P4mm associated with point group 4mm. Figure 1 shows that 4mm goes directly to mm2 but the latter can occur in two crystallographically different orientations depending on whether the mirror planes (100) and (010) are retained or alternatively the planes (110) and $\left(1\bar{1}0\right)$. We thus expect to obtain two space groups associated with mm2 from P4mm and figure 5 shows that these are Pmm2 and Cmm2. The former corresponds to retention of (100) and (010) mirror planes and of the original coordinate axes. The latter corresponds to retention of (110) and $\left(1\bar{1}0\right)$ mirror planes and to a change of cell to a C-face-centered cell of twice the volume with its [100] axis along $\left[1\bar{1}0\right]$ of the original cell. Pmm2 can go to Pm with point group m in two ways and then to P1, the final space group with no symmetry to which all non-centrosymmetric space groups must ultimately reduce. Pmm2 can alternatively go to P2 and then to P1. Returning to the other branch coming from P4mm, we see that Cmm2 can go either to Cm and then to P1, or to P2 and then P1.

The behavior of a set of Wyckoff positions under homogeneous strain is important in determining whether internal friction can occur [3] and may be relevant to the subjects mentioned in the introduction. We have noted in the previous section that the set of *N* general positions of an initial space group always map onto *N*/*n* sets of general positions in the lower space group. The mapping of a set of special positions of the starting space group onto sets of special and/or general positions of the lower space group is more complicated. In particular it should be noted that splitting into unequal subsets is possible for some special orientations of the strain ellipsoid relative to symmetry elements of the point group [*3*]. For example the oxygen ions in corundum (*α*-Al_2_O_3_, $R\bar{3}c$) occupy a set of positions designated “e” by Wyckoff and located on diad axes. The other symmetry operations generate a set of six equivalent e-type positions per primitive cell. Tensile strain parallel to an a-axis retains the centers of symmetry, the diad axes parallel to, and the glide planes perpendicular to the direction of the tensile strain. The set of six initially equivalent oxygen positions then splits into one subset of four and another of two equivalent positions. A table of the mapping of special positions onto positions of subgroups has not yet been completed.

## Footnotes

1 Italicized figures in brackets indicate the literature references at the end of this paper.
